# ALDH1A1 expression is associated with poor differentiation, ‘right-sidedness’ and poor survival in human colorectal cancer

**DOI:** 10.1371/journal.pone.0205536

**Published:** 2018-10-11

**Authors:** Lizet M. van der Waals, Inne H. M. Borel Rinkes, Onno Kranenburg

**Affiliations:** Laboratory Translational Oncology, UMC Utrecht, Utrecht, The Netherlands; University of New Mexico, UNITED STATES

## Abstract

**Background:**

Aldehyde dehydrogenase 1A1 (ALDH1A1) encodes an enzyme that oxidizes aldehydes to their corresponding carboxylic acids. In colorectal cancer ALDH1A1 marks cancer stem cells and plays putative roles in tumor progression and drug resistance. However, the potential value of ALDH1A1 as a diagnostic marker or target for therapy remains unclear. Here, we have analyzed ALDH1A1 mRNA and protein levels in relation to clinical, histopathological and molecular tumor features in large series of human colorectal cancer.

**Methods:**

ALDH1A1 protein levels were determined by immunohistochemistry in a series of primary colorectal tumors and their corresponding liver metastases (n = 158). ALDH1A1 mRNA levels were analyzed in several large patient cohorts of colorectal cancer. ALDH1A1 mRNA and protein levels were then related to overall survival and to clinical, histopathological and molecular tumor features.

**Results:**

High levels of ALDH1A1 were associated with a poorly differentiated histology and a right-sided tumor location, but not to a mesenchymal-like molecular subtype. Liver metastases contained significantly higher levels of ALDH1A1 compared to the corresponding primary tumors. Radio- and/or chemotherapy prior to tumor resection was associated with increased ALDH1A1 levels regardless of the molecular subtype. Finally, ALDH1A1 protein expression in primary tumors and metastases correlated with shorter overall survival.

**Conclusions:**

ALDH1A1 expression is associated with features of poor prognosis, including a poorly differentiated histology and ‘right-sidedness’ of the primary tumor, and with shorter overall survival. ALDH1A1 is also highly expressed in therapy-surviving tumors and in liver metastases. These results warrant further research into the potential value of targeting ALDH1A1 in order to improve the efficacy of standard treatment and thereby preventing tumor recurrence.

## Introduction

About 20–25% of colorectal cancer (CRC) patients present with metastatic disease [1, 2] and almost 50% of all CRC patients will develop metastases [2]. The prognosis for metastatic CRC (mCRC) remains poor, with a medium overall survival (OS) up to approximately 30 months after chemotherapy combined with targeted agents [3, 4]. New treatment approaches that effectively target functional markers of poor prognosis and metastatic disease are urgently needed.

Aldehyde dehydrogenase 1A1 (ALDH1A1) (NX_P00352) is a member of the ALDH superfamily, of which nineteen different ALDH functional genes have been characterized to date [5–8]. The ALDH gene family represents a diverse group of proteins, that detoxify endogenous and exogenous aldehydes through NAD(P)+-dependent oxidation [9]. Different biological functions have been attributed to the different ALDH family members [5–8]. The cytosolic enzyme ALDH1A1 is involved in the catalysis of retinaldehyde to retinoic acid (RA) [7, 8]. RA binds to the nuclear RA receptors, RARα and RXR, and thereby regulates transcriptional activity of genes involved in multiple important processes including proliferation, differentiation and apoptosis [7, 8]. Furthermore, ALDH1 is able to protect cancer stem cells (SCs) against high levels of reactive oxygen species [7, 8]. In addition, ALDH(1A1) expression is possibly associated with resistance to radiotherapy [10, 11] and chemotherapeutics commonly used to treat CRC [12, 13]. Previous research shows that ALDH1A1 has tumorigenic and metastasis-promoting functions [7, 8]. The ALDH1A1 isozyme has historically been the best studied ALDH family member linked to SCs [8] and the ALDH1A1 isozyme specifically has also been linked to colon cancer stem cells (SCs) [14]. Although ALDH1A1 might thus be involved in CRC, its relation to features of poor prognosis in CRC, including a poorly differentiated histology [15, 16], ‘right-sidedness’ of the primary tumor [17], presence of metastases [3, 4] and a mesenchymal (MES)-like phenotype [18] is not clear. Previous studies looking into ALDH1A1 levels and CRC survival showed contradictory data [19–22].

We therefore conducted immunohistochemical analyses of ALDH1A1 levels in patient-derived primary CRC tissue and corresponding liver metastases in relation to tumor characteristics and clinical parameters. The aim of this study was to assess whether ALDH1A1 could serve as a biomarker for CRC biology and/or clinical outcome. Our results show that high ALDH1A1 levels are linked to a shorter OS and to tumor characteristics that are associated with a shorter survival in CRC.

## Materials and methods

### Patients and tissues

The patient cohort was described in [23]. In short, 158 mCRC patients that underwent a resection for colorectal liver metastases (CRLM) at the University Medical Center Utrecht were included in a prospective database. Clinicopathological data was available from 129 patients. 86 males and 43 females were included. Mean age was 62 years at time of surgery for CRLM. The study was approved by the medical ethical committee University Medical Center Utrecht (protocol #09–145). Tumor tissue and anonymous patient data was obtained in accordance with relevant guidelines and regulations on human experimentation. Written informed consent was obtained from all participants and/or their legal guardian(s).

### Tissue microarray construction

A triple-core tissue microarray (TMA) of primary CRC tumors and corresponding CRLM was constructed as described before [23].

### Immunohistochemistry

Immunohistochemical staining was done on 4 μm sections of formalin-fixed paraffin-embedded TMA samples as described previously [24]. In short, antigen retrieval was performed by boiling samples for 20 minutes in Citrate Buffer pH 6.0. Mouse monoclonal antibodies specific for ALDH1A1 (clone 44, BD Biosciences, San Jose, CA, USA, 611195, 1:500) were incubated for 1 h at room temperature. Subsequently, the secondary antibodies BrightVision poly-HRP-conjugated anti-mouse IgG (ImmunoLogic, Duiven, The Netherlands, ready to use) were incubated for 30 min at room temperature. All sections were developed with diaminobenzidine for 10 min, followed by a hematoxylin counterstaining.

### Evaluation of antibody specificity

**Western blotting**—Decreasing amounts of recombinant ALDH1A1 (Sino Biological, Wayne, PA, USA, 11388-H07E) and recombinant ALDH1A3 (Sino Biological, Wayne, PA, USA, 11636-H07E) dissolved in sterile H_2_O were loaded on 12% tris-glycine SDS-polyacrylamide gels. Proteins were separated by electrophoresis, and transferred to PVDF membranes, which were blocked in TBST 5% milk (Merck) for 1 h at room temperature. Membranes were probed overnight at 4°C with primary mouse monoclonal antibodies specific for ALDH1A1 (clone 44, BD Biosciences, San Jose, CA, USA, 611195, 1:625) washed in TBST, incubated with secondary antibodies goat-anti-mouse (Dako, P0447, 1:2000) conjugated to horseradish peroxidase at room temperature for 1 h, and developed using ECL Western Blotting Reagents (GE Healthcare, Chicago, IL, USA). During processing, images were converted to grayscale and Photoshop levels adjustment was used to improve tonal quality.

**Immunohistochemistry**—Colon and CRC tissue slides were used to evaluate ALDH1A1 staining pattern. Kidney tissue was used as positive control based on previously observed ALDH1A1 positive protein staining patterns in kidney tissue available from the Human Protein Altas (https://www.proteinatlas.org/ENSG00000165092-ALDH1A1/tissue/kidney v18 Human Protein Atlas). Prostate cancer tissue was used as negative control based on protein expression data available from the Human Protein Atlas (https://www.proteinatlas.org/ENSG00000165092-ALDH1A1/pathology v18 Human Protein Atlas) showing that ALDH1A1 immunoreactivity in this tumor type is almost always absent. To check for nonspecific binding of the secondary antibody, tissue sections were incubated without primary antibodies in the presence of the secondary antibody.

### Evaluation of staining

Scoring was performed by consensus of two investigators (L.M.W. and K.V.) blinded to clinicopathological data, treatment status and epithelial (EPI)- versus MES-like subtype. Damaged and empty TMA cores and those not containing cancer cells were excluded. Staining within clearly necrotic regions was ignored and only cytoplasmic immunoreactivity was considered. The percentage of positive tumor cells was scored per intensity, calculating H-scores [25] as follows (1*(% cells with intensity 1) + 2*(% cells with intensity 2) + 3*(% cells with intensity 3) (S1 Fig). The mean H-scores of 3 cores for each primary tumor specimen or metastatic specimen per patient were calculated. Nuclear ALDH1A1 staining was separately scored in addition to cytoplasmic staining as negative or positive and associated with OS. TMA raw data are provided in S1 Table.

### Subgroups

Tumors located in the descending colon, sigmoid, rectosigmoid or rectum were referred to as left-sided colorectal carcinomas, unless indicated otherwise. Right-sided colon carcinomas included tumors located in the ascending colon and caecum. Given the differences in embryonic origin between the proximal and distal part of the transverse colon [17], the different classifications used [17], and the low number of these tumors (n = 3) we decided to exclude these tumors from our tumor location subanalyses. Tumor tissue was stratified according to their treatment status as described before [24]. Immunohistochemistry-based molecular classification of all tumors within the TMA into EPI-like and MES-like CRC molecular subtypes was performed in the original study [23].

### Bioinformatic analyses

All bioinformatic analyses were performed using the R2 Genomics Analysis and Visualization Platform (https://hgserver1.amc.nl/cgi-bin/r2/main.cgi registration required). Gene expression data were obtained from a large primary colon dataset (GSE39582) [26], a composite CRC cohort dataset (GSE14333, GSE17536, GSE17537) [27, 28], and two independent datasets containing expression data from CRC liver metastases Array Express (E-TABM-1112) [29] and GSE5851 [30]. Furthermore, a small dataset that contains mRNA data from normal colorectal mucosa obtained from patients diagnosed with colorectal adenomas was used (GSE8671) [31].

### Statistics

All analyses were carried out using IBM SPSS statistics version 23 or 25 (IBM corp., Armonk, NY), GraphPad Prism version 7.0 for Windows (GraphPad Software, La Jolla, California, USA) and Excel version 2013. GraphPad Prism and SPSS were used for graphical presentation of the data. p < 0.05 (two-tailed) was considered statistically significant. All clinicopathological characteristics of the patients were assessed using a Chi-square Test and Independent-samples T-test for the continuous variable age. As the immunohistochemical expression data was right-skewed, we log-transformed the data before analyzing and comparing mean differences in protein levels. Results were reported on the original scale after back transformation, yielding geometric means. To account for values of zero using this approach, 0.5 was added to all data-points before log-transformation. A one-way ANOVA followed by a Fisher’s LSD test was performed on the log-transformed data to compare ALDH1A1 levels after stratification according to differentiation status or treatment status in EPI-like and MES-like tumors. A paired T-test was applied to the log-transformed data to compare ALDH1A1 levels in primary CRC samples and corresponding CRLM before and after stratification according to tumor location. An Independent-samples T-test was performed to compare ALDH1A1 levels in left-sided against right-sided tumors, in treated versus untreated tumor samples, and in left-sided against right-sided tumors within primary or metastatic tissue. For survival analysis, OS was measured from time of liver surgery until death or end of 10 year follow-up. Kaplan-Meier survival analyses were performed and survival curves were compared between the different groups by log-rank tests. The Cox proportional hazards regression model was used to evaluate the effect of ALDH1A1 protein expression in addition to age, gender, tumor depth (T stage), lymph node status (N stage), presence of distant metastases (M stage), differentiation status, treatment status, and number of liver metastases.

## Results

To assess whether ALDH1A1 expression was related to various clinical and histopathological features in human CRC we made use of a TMA consisting of 158 paired samples of primary tumors and their corresponding metastases. ALDH1A1 protein levels were measured by immunohistochemistry.

To validate the specificity of the mouse monoclonal antibody against ALDH1A1, we first checked whether the antibody recognizes recombinant ALDH1A1 using Western blotting (Panel A in S2 Fig). Our results show a clear ALDH1A1 band at the correct height of 55 kDa (Panel A in S2 Fig). Importantly, the ALDH1A1 antibody did not recognize the ALDH1 isozyme ALDH1A3, which amino acid sequence resembles ALDH1A1 the most (Panel A in S2 Fig). We next evaluated the immunohistochemical staining pattern of the antibody against ALDH1A1 in normal colon tissue. As expected, the intestinal crypts containing SCs were slightly more positive (Panel B in S2 Fig). In addition, ALDH1A1 was highly expressed in CRC tumor tissue whereas normal colon tissue was mainly negative (Panel B in S2 Fig). In line with the staining pattern of two other ALDH1A1 antibodies shown by the Human Protein Atlas (https://www.proteinatlas.org/ENSG00000165092-ALDH1A1/tissue/kidney#imid_5585676 v18 Human Protein Atlas), cells in kidney tubules where positive whereas cells in glomeruli were negative (Panel B in S2 Fig). Prostate cancer tissue was used as negative control and showed no immunoreactivity (Panel B in S2 Fig). These results, in addition to a previous study [32], confirmed the specificity of the ALDH1A1 staining.

**Fig 1:**
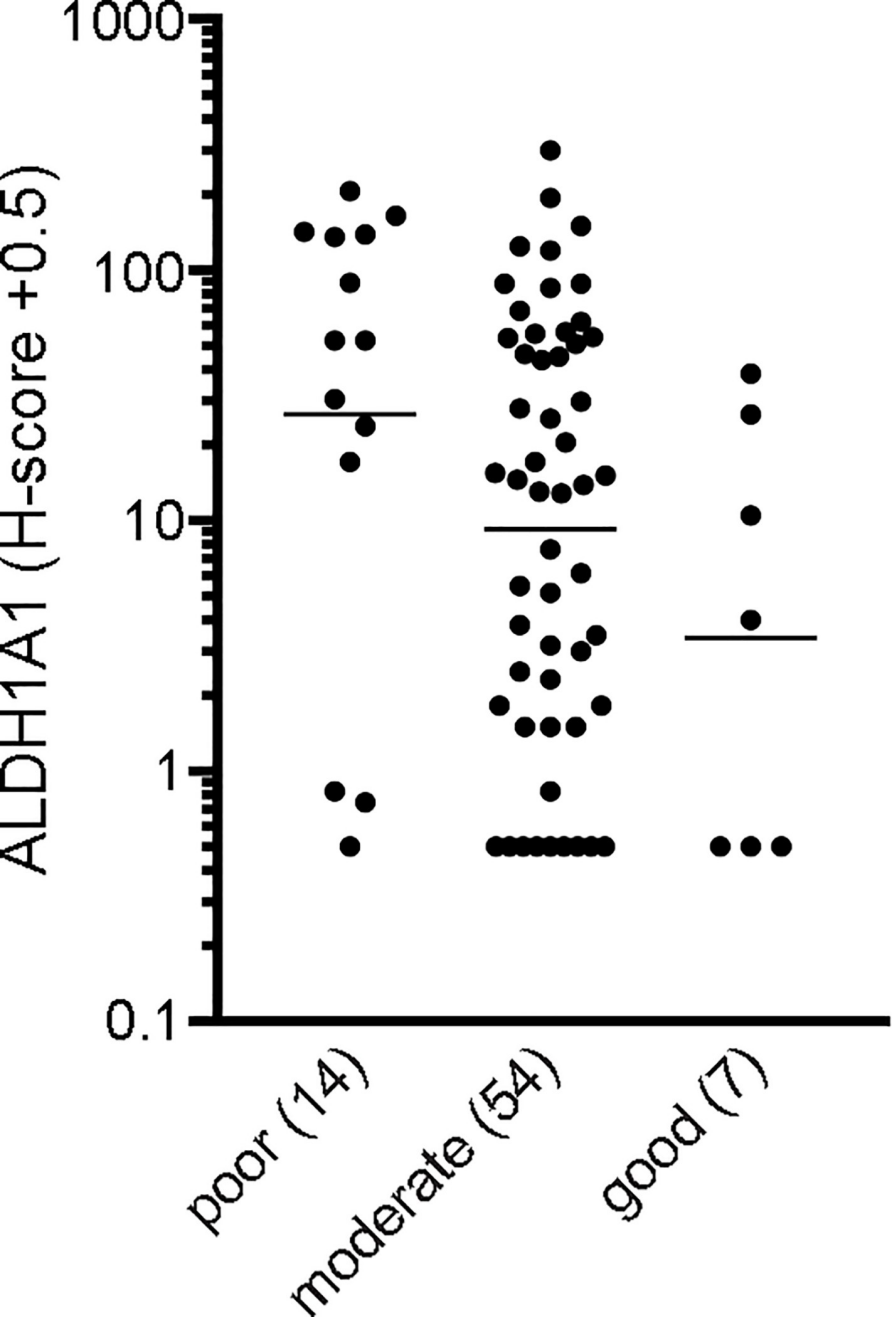
High ALDH1A1 levels are associated with poorly differentiated tumors. Scatter dot plot showing protein levels of ALDH1A1 in untreated primary colorectal cancer tumors stratified according to differentiation grade. A one-way ANOVA was applied to the log-transformed data to compare groups (p > 0.05). The geometric mean is shown.

After validation of the ALDH1A1 antibody, we next correlated ALDH1A1 expression to tumor differentiation. We found that ALDH1A1 protein levels in primary, non-treated CRC increased with a progressive loss of histological differentiation. (Fig 1: p = 0.069 poor vs good).

**Fig 2:**
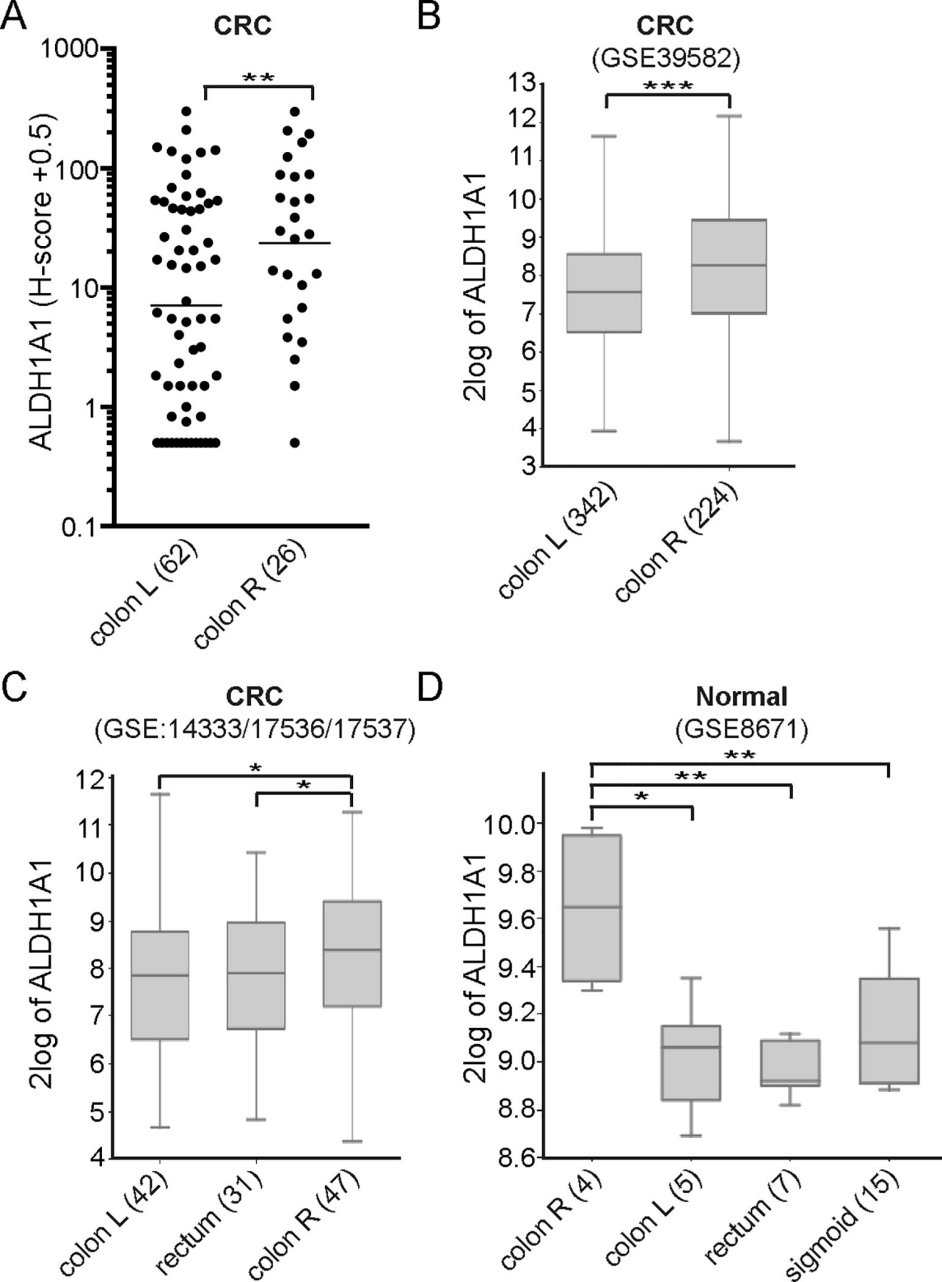
ALDH1A1 expression is associated with right-sided colon carcinomas. Scatter dot plot and box plots showing ALDH1A1 protein (A) or mRNA (B-D) levels in untreated primary colorectal cancer (CRC) tumors (A-C) or normal tissue (D) stratified according to tumor localization. Gene expression data were obtained from a large primary colon dataset [26] (B), a composite CRC cohort dataset [27, 28] (C) and a dataset containing expression data from normal colorectal mucosa obtained from patients diagnosed with colorectal adenomas [31] (D). (A) An unpaired T-test was applied to the log-transformed data to compare groups. The geometric mean is shown. (B-D) The box represents the median and middle 50% of the scores. The upper and lower whiskers represent scores outside the middle 50% (one-way ANOVA). CRC, colorectal cancer; L, left; R, right. *, p ≤ 0.05; **, p ≤ 0.01; ***, p ≤ 0.001.

Primary tumor localization is related to clinical outcome in mCRC [17]. We found that levels of ALDH1A1 in untreated right-sided tumors were significantly higher (3.8-fold) when compared to left-sided tumors (Fig 2A). Further stratification of left-sided tumors showed that levels of ALDH1A1 are lower in all parts of the left-sided colon (descending colon, rectum or sigmoid) when compared to right-sided tumors (Panel A in S3 Fig). There was a trend towards higher levels of ALDH1A1 in right-sided tumors compared to rectal tumors (p = 0.072) (Panel A in S3 Fig). Interestingly, if treatment-exposed rectal tumors were included in the analysis this difference almost completely disappeared (Panel B in S3 Fig). In addition to increased ALDH1A1 protein levels in right-sided tumors we also found that ALDH1A1 mRNA levels were significantly higher in right-sided tumors in two independent CRC cohorts [26–28] (Fig 2B and 2C). We reasoned that the tissues from which the tumors originated may be characterized by differential ALDH1A1 expression. Indeed, we found that ALDH1A1 mRNA levels were significantly higher in normal ‘healthy’ right-sided colon tissue when compared to ‘healthy’ left-sided colon tissue in a cohort of patients diagnosed with colorectal adenomas [31].

**Fig 3:**
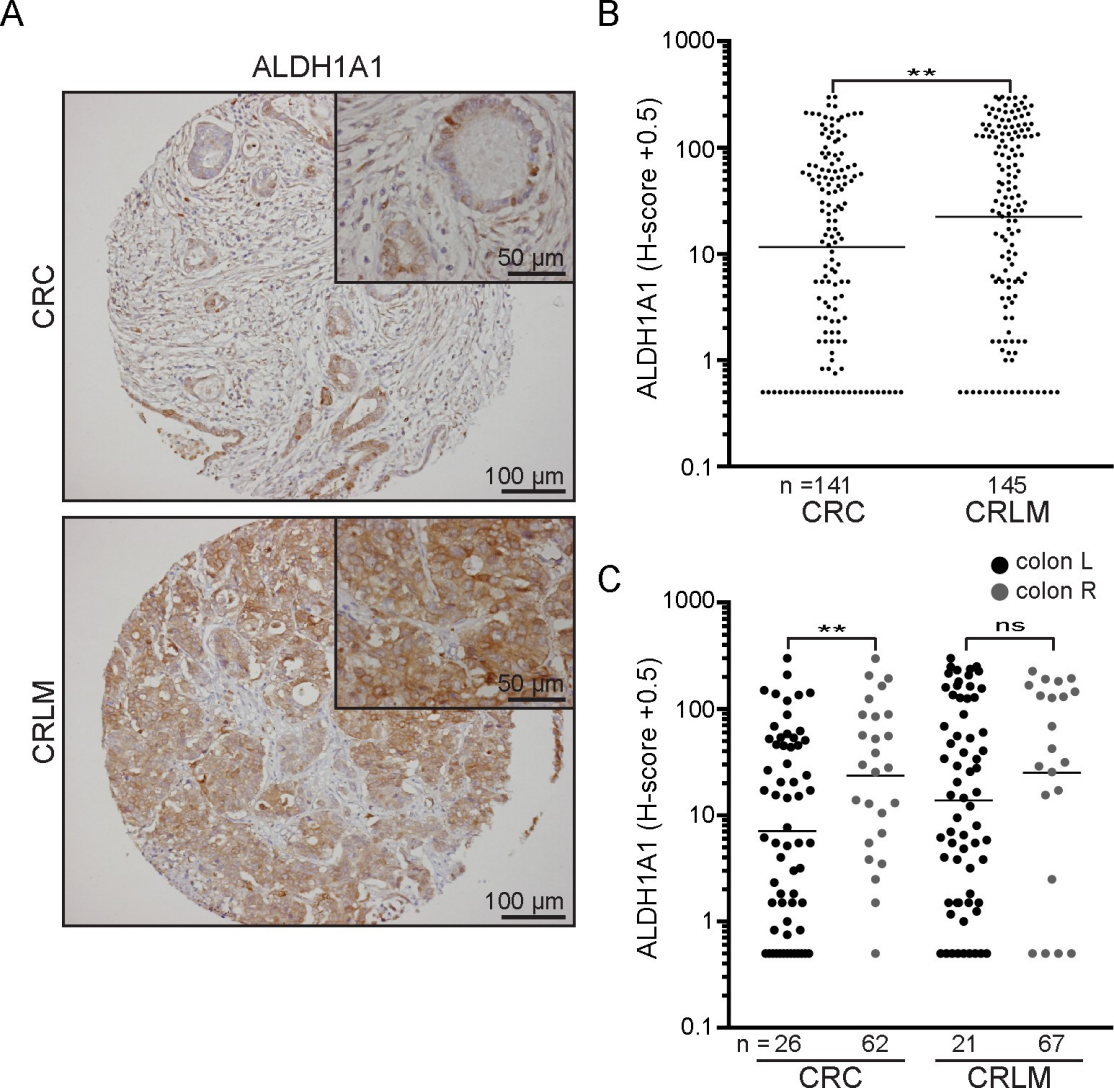
ALDH1A1 levels are increased in liver metastases. (A) Representative images of immunohistochemical expression of ALDH1A1 in primary colorectal cancer (CRC) samples and corresponding colorectal liver metastases (CRLM). x10 microscope objective whole TMA tissue core and x40 microscope objective inset shown. (B) Scatter dot plot showing quantification of ALDH1A1 levels in CRC tumors versus CRLM. A paired T-test was applied to the log-transformed data to compare ALDH1A1 expression as continuous variable in CRC versus CRLM. (C) Scatter dot plot showing quantification of ALDH1A1 levels in primary CRC and CRLM, left-sided- versus right-sided tumors. An unpaired T-test was applied to the log-transformed data to compare ALDH1A1 levels within primary CRC tumors and metastases. A paired T-test was used to analyze levels of ALDH1A1 in either left- or right-sided primary tumors and corresponding metastases (p > 0.05). ns, p > 0.05; **, p ≤ 0.01. The geometric mean is shown. CRC, colorectal cancer; CRLM, colorectal liver metastases; L, left-sided; R, right-sided.

We next tested whether levels of ALDH1A1 were different between primary tumors and paired liver metastases. ALDH1A1 levels were significantly increased (2-fold) in liver metastases compared to corresponding primary tumors (Fig 3A and 3B and S4 Fig). We next stratified primary tumors and metastases according to their primary tumor location. Interestingly, levels of ALDH1A1 mainly varied between metastatic tissue and primary left-sided tumors (Fig 3C).). The high levels of ALDH1A1 in right-sided tumors were maintained, but did not further increase in their metastases (Fig 3C).

**Fig 4:**
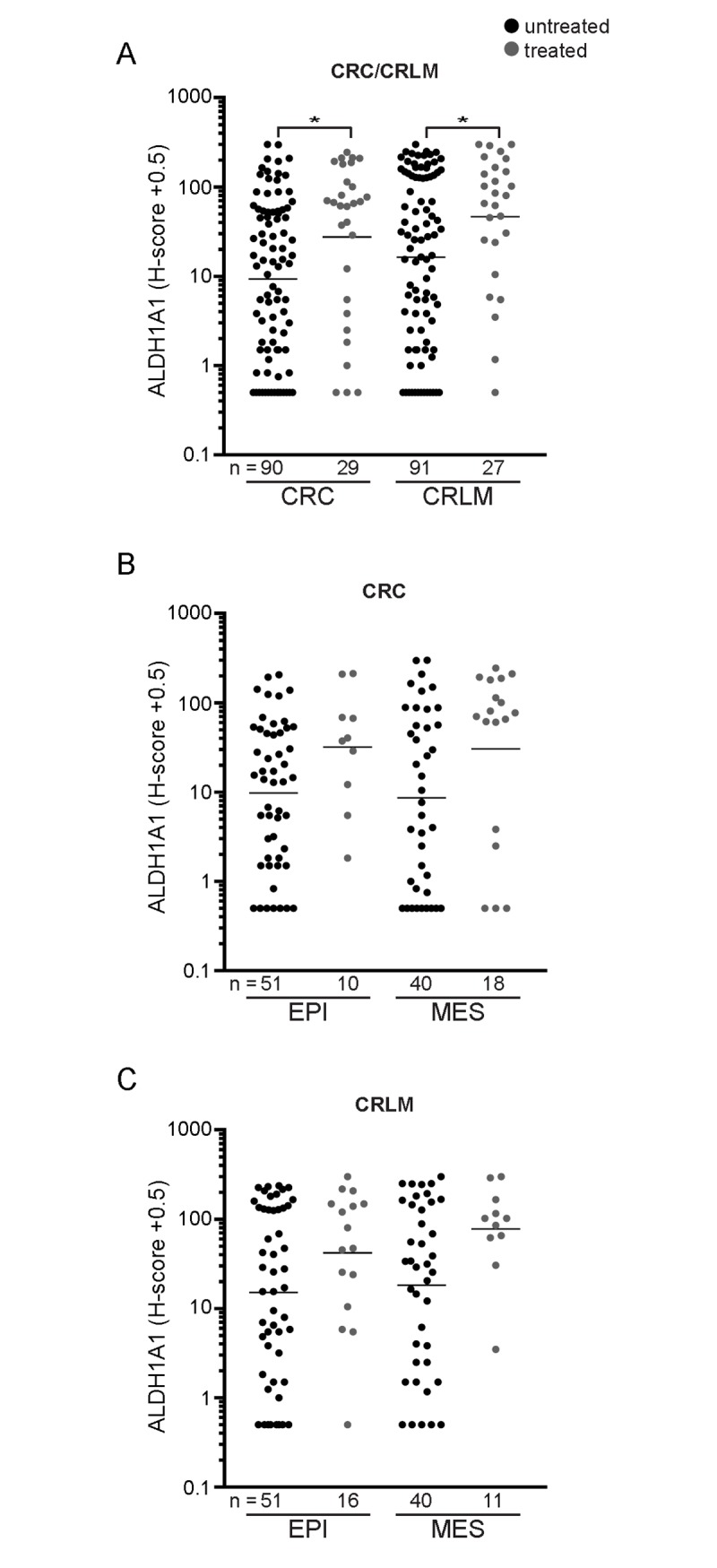
ALDH1A1 levels are higher in therapy-exposed colorectal cancer tissue. (A) Scatter dot plot showing quantification of ALDH1A1 levels in primary colorectal cancer (CRC) and colorectal liver metastases (CRLM), untreated versus treated tumors. An unpaired T-test was applied to the log-transformed data to compare untreated versus treated primary tumors or metastases. Levels of ALDH1A1 in primary CRC tumors (B) and CRLM (C) stratified according to treatment status and CRC subtype. A one-way ANOVA was applied to the log-transformed data to compare groups (ns, p > 0.05). *, p ≤ 0.05. The geometric mean is shown. CRC, colorectal cancer; CRLM, colorectal liver metastases; EPI, epithelial; MES, mesenchymal.

ALDH1A1 is known to provide cellular protection by detoxification of cytotoxic drugs [8]. Therefore, we speculated that the increased levels of ALDH1A1 in CRLM could be a consequence of differences in treatment status between primary CRC tumors and their corresponding liver metastases. To test this, we stratified primary tumors and metastases according to their treatment status. This revealed that treatment prior to tumor resection was associated with significantly higher levels of ALDH1A1, both in primary tumors and metastases (Fig 4A).

Interestingly, ALDH1A1 levels were higher in previously untreated metastases compared to untreated primary tumors (Fig 4A), suggesting that prior treatment per se cannot explain the higher levels of ALDH1A1 in liver metastases compared to primary tumors. Of interest, previous research shows that ALDH1A1 is a target of the nuclear receptor pregnane X receptor [33], also known as nuclear receptor subfamily 1 group I member 2 (NR1I2) (NX_O75469). NR1I2 is involved in xenobiotic metabolism and expressed in colon cancer SCs [33]. The presence of xenobiotics within the liver microenvironment might thus be (partly) responsible for the higher levels of ALDH1A1 observed in liver metastases. To test this hypothesis, we analyzed whether gene expression of ALDH1A1 is positively associated with gene expression of NR1I2 in two independent CRC cohorts containing primary tumor samples [26–28] versus two cohorts containing metastatic tissue [29, 30]. Our data shows a significant positive correlation between ALDH1A1 expression and expression of NR1I2 in CRC liver metastases, but not in primary CRC (S5 Fig). Interestingly, liver metastases with high expression of both ALDH1A1 and NR1I2 also expressed high levels of the KEGG pathway genes (www.KEGG.jp) involved in xenobiotics metabolism.

We next used immunohistochemistry-based classification into molecular subtypes [34] to discriminate between EPI-like and MES-like tumors. MES-like tumors are associated with poor prognosis [18]. We found that untreated primary tumors and metastases of the MES-like subtype did not show significantly increased levels of ALDH1A1 compared to the EPI-like tumors (Fig 4B and 4C). Interestingly, protein levels of ALDH1A1 were higher in both MES-like and EPI-like tumors that received prior treatment (Fig 4B and 4C), indicating that the effect of prior treatment on ALDH1A1 expression is unrelated to molecular subtype.

**Fig 5:**
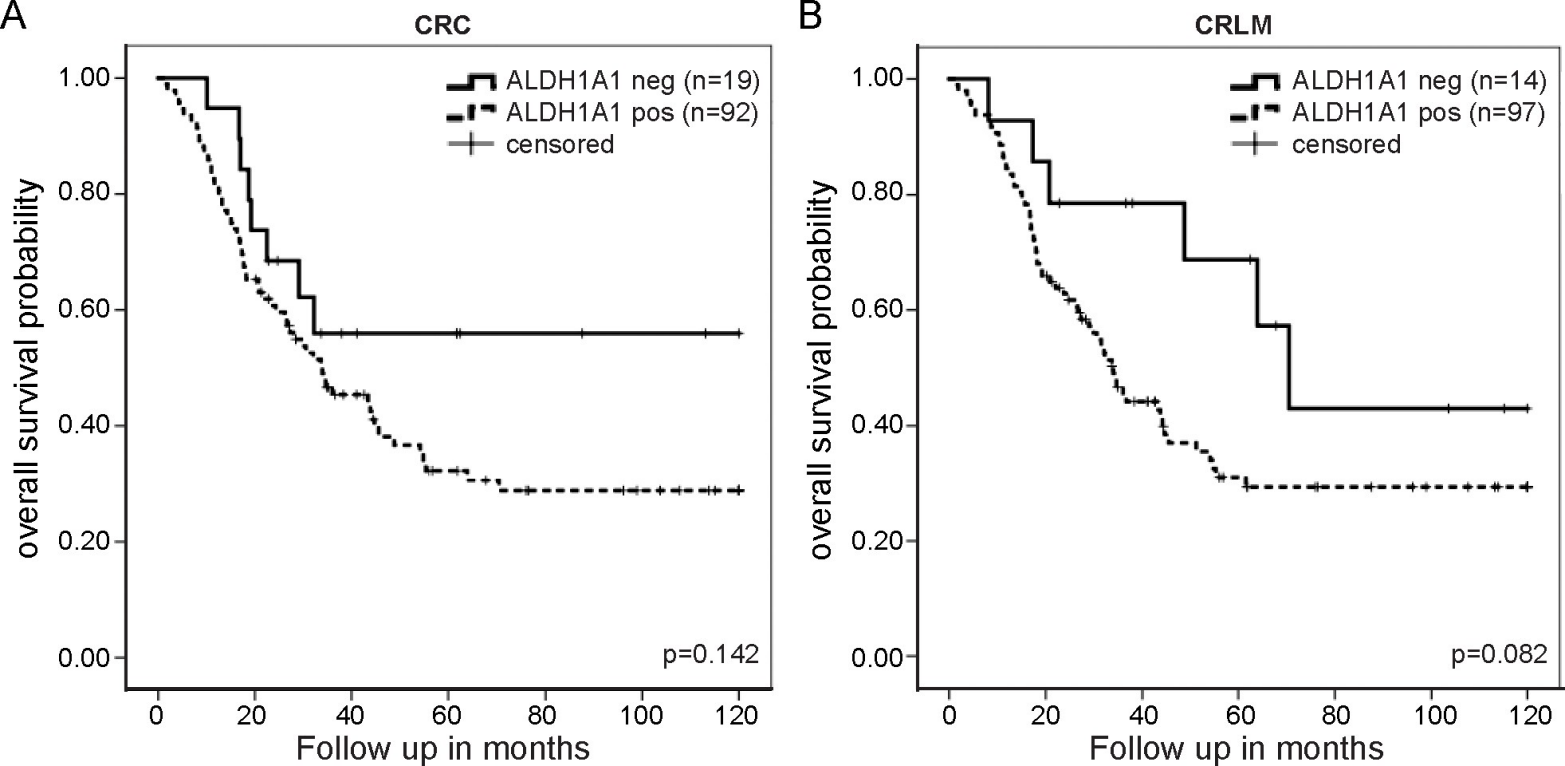
ALDH1A1 positivity identifies a group of poor-prognostic primary tumors and metastases. (A) Kaplan-Meier curves showing differences in overall survival between ALDH1A1-positive and ALDH1A1-negative primary colorectal cancer tumors. Significance was tested using the log-rank test. (B) Kaplan-Meier curves showing differences in overall survival between ALDH1A1-positive and ALDH1A1-negative colorectal liver metastases. Significance was tested using the log-rank test. CRC, colorectal cancer; CRLM, colorectal liver metastases; Neg, negative; Pos, positive.

Studies assessing the link between ALDH1A1 expression in CRC and prognosis are not conclusive [19–22]. This prompted us to analyze OS in relation to ALDH1A1 immunohistochemical staining. We stratified patients according to their H-score based on ALDH1A1 immunohistochemistry (H-score = 0 versus H-score>0). An absolute cut-off for evaluating immunohistochemical staining (i.e. negative versus positive) will generally lead to low inter- and intra-observer variability and a wide applicability of the method. Patients with ALDH1A1-positive primary tumors and liver metastases had a shorter OS than patients with ALDH1A1-negative tumors (Fig 5A and 5B). The hazard ratio after multivariate analysis is 1.442 (95% confidence interval [95% CI] 0.582–3.575, p = 0.429) for ALDH1A1-positive primary tumors and 2.367 (95% CI 0.939–5.969, p = 0.068) for ALDH1A1-positive liver metastases. In line with a previous report [22], we also observed nuclear expression of ALDH1A1. Interestingly, presence of nuclear ALDH1A1 was also associated with a shorter OS (S6 Fig).

Of interest, all right-sided primary tumors, except one, were characterized by a positive ALDH1A1 status (Table 1). For statistical analysis, we further focused on the left-sided tumors and found that a positive ALDH1A1 status was related to a shorter OS (Panel A in S7 Fig). In addition, the majority of all metastases derived from right-sided primary tumors was also characterized by a positive ALDH1A1 status (Table 1). Finally, a positive ALDH1A1 status in metastases derived from left-sided tumors was associated with a shorter OS (Panel B in S7 Fig).

**Table 1:** Characteristics of patients with a positive or negative ALDH1A1 status.

	CRC (n = 111)					CRLM (n = 111)				
	Neg		Pos			Neg		Pos		
	n = 19	%	n = 92	%	p-value	n = 14	%	n = 97	%	p-value
**Mean age**										
years, range	59	(42–80)	62	(37–83)	0.274	61	(47–72)	61	(37–82)	0.849
**Sex**										
Male	15	79	58	63	0.183	11	79	63	65	0.312
Female	4	21	34	37		3	21	34	35	
**Onset**										
Synchronous Metachronous	-	-	-	-	-	6	43	56	58	0.295
						8	57	41	42	
**Site of CRC**										
Right colon Left colon Sigmoid Rectum Transverse colon	1	5	23	25	0.007	4	29	16	17	0.084
	3	16	8	9		2	14	9	9	
	8	42	29	32		6	43	29	30	
	5	26	31	34		2	14	38	40	
	2	11	1	1		0	0	4	4	
**Invasion depth**										
T2 T3 T4 Unknown	1	5	7	8	0.944	-	-	-	-	-
	14	74	67	73						
	4	21	17	18						
	0	0	1	1						
**Lymph node status**										
N0 N1 N2 Unknown	6	32	39	39	0.238	-	-	-	-	-
	6	32	37	37						
	6	32	15	15						
	1	5	1	1						
**Dukes classification**										
B1 B2 C1 C2 D	0	0	4	4	0.874	-	-	-	-	-
	3	16	15	16						
	0	0	1	1						
	4	21	21	23						
	12	63	51	55						
**Differentiation**										
Good Moderate Poor Unknown	1	5	12	13	0.476	-	-	-	-	-
	12	63	60	65						
	3	16	6	7						
	3	16	14	15						
**Treatment status**										
Treated Untreated	16	84	68	74	0.341	13	93	71	73	0.109
	3	16	24	26		1	7	26	27	

All Chi-square Test (categorical data) except for the continuous variable age at surgery (Independent-samples T-test). CRC, colorectal cancer; CRLM, colorectal liver metastases; Neg, negative; Pos, positive.

## Discussion

In the present study we have shown that ALDH1A1 is associated with poorly differentiated CRC tumors. This is in line with a meta-analysis in head and neck squamous cell carcinoma [35], showing that high ALDH1A1 expression was associated with poorly differentiated tumors. Moreover, Lugli et al. [19] showed that ALDH1A1 expression was related to poorly differentiated tumors in CRC. The association of ALDH1A expression with poor differentiation may reflect the CSC-like nature of ALDH1-expressing tumor cells [6–8, 36, 37] in both CRC and head and neck squamous cell carcinoma.

Right-sided primary tumor location is a confirmed negative prognostic variable in CRC [17]. Although the underlying mechanisms are not clear, differences in the microbiome, clinical characteristics, chromosomal and molecular characteristics may play a role in the poor prognosis of these tumors [17]. We show that right-sided tumors, and also the metastases derived from such tumors, express higher levels of ALDH1A1 than left-sided tumors. Primary tumor location should therefore be taken into consideration in the design of clinical studies testing ALDH1(A1)-targeting drugs.

Treatment regimens effectively targeting CRLM are an important unmet medical need. We observed that ALDH1A1 protein levels were significantly higher in liver metastases than in primary tumors from which they were derived and highest in metastases derived from right-sided tumors. Interestingly, knockdown of ALDH1A1 in breast cancer cells reduced *in vitro* and *in vivo* breast cancer metastatic ability [38]. In addition, the percentage of positive ALDH1A1-expressing cells was significantly increased in lymph node metastases of oropharyngeal squamous cell carcinoma [39]. ALDH1A1 levels were increased in CRC lymph node metastases, although not significantly [20]. We further showed that even after stratification for treatment status, ALDH1A1 was higher in liver metastases than in primary tumors. This indicates that factors such as the microenvironment [40] and/or intrinsic differences between metastasis-competent and -incompetent subclones may underlie the different levels of ALDH1A1 in primary tumors and paired liver metastases. Indeed, we showed that there is a positive relationship between mRNA levels of the nuclear receptor NR1I2, which might become activated upon exposure to xenobiotics present in the liver microenvironment, and its downstream target ALDH1A1 [33] in two independent cohorts containing tumor tissue derived from CRLM.

ALDH1A1 levels were significantly higher in patients that received radio- and/or chemotherapy prior to tumor resection. This indicates that tumor cells increase their levels of ALDH1A1 in response to treatment and/or that high levels of ALDH1A1 make tumor cells less responsive to treatment, resulting in their therapy-induced selection. That treatment increases ALDH1A1 positivity has been shown in breast cancer biopsies taken pre- and post-chemotherapy [41], although this occurred in a minority of samples. Interestingly, in the same study, a pathological complete response was more often observed in ALDH1A1-negative tumors [41]. Breast cancer cells that demonstrate high activity of ALDH and high expression of CD44 are more resistant to both chemotherapy (doxorubicin/paclitaxel) and radiation [10]. Moreover, knockdown of ALDH1A1 sensitizes breast cancer cells to chemotherapy and radiation [38]. Additionally, cisplatin exposure has been shown to select for resistant ALDH1A1-positive cells in non-small cell lung cancer [12]. Another relatively small study observed that a poor response to neoadjuvant chemotherapy is related to an increase in ALDH1A1 levels after therapy in breast cancer patients [42]. Treatment of CRC cell lines with 5-fluorouracil increased the ALDH+ cell fraction as determined with the Aldefluor assay [13]. Moreover, ALDH1A1 mRNA levels were increased in tumor tissue obtained from colon carcinoma tumor-bearing mice treated with a combination of leucovorin, 5-fluorouracil, and irinotecan (FOLFIRI) compared to non-treated mice [33]. Our data and previous work thus warrants further research into targeting ALDH1A1 to prevent therapy resistance and tumor recurrence after treatment. Interestingly, new ALDH1(A1)-targeting agents are under development and clinical trials are being conducted [6, 7].

In conclusion, ALDH1A1 expression in primary tumors and liver metastases identifies a subset of CRC tumors with poorly differentiated histology, right-sided tumor location and poor prognosis. Prior treatment further increases ALDH1A1 protein levels. Future studies should evaluate the potential value of targeting ALDH1A1 in subgroups of CRC patients. If successful this would imply a potential role for such therapeutics in improving treatment response and OS in poor-prognostic CRC patients.

## Supporting information

S1 Fig Representative pictures as example of immunohistochemical intensity scoring of ALDH1A1 levels. Images of the different staining intensities: 0 (negative), 1 (weak), 2 (moderate) or 3 (strong) are shown. I = intensity. Scale bar: 100 μm (x10 microscope objective). Inset shows higher magnification.
(PDF)

S2 Fig Evaluation of ALDH1A1 antibody staining specificity. (A) Decreasing amounts of either recombinant ALDH1A1 (rALDH1A1) or rALDH1A3 were loaded to evaluate ALDH1A1 antibody specificity using Western blotting. (B) Immunohistochemical staining pattern of the antibody against ALDH1A1 in normal colon tissue, CRC tumor tissue, kidney and prostate cancer (negative control). White arrows indicate ALDH1A1 immunoreactivity in intestinal crypts (colon tissue) or absence of ALDH1A1 staining in kidney glomeruli. Scale bar: 100 μm (x10 microscope objective).
(PDF)

S3 Fig ALDH1A1 levels in primary tumors stratified according to tumor localization. (A) Scatter dot plot showing protein levels of ALDH1A1 in untreated primary colorectal cancer (CRC) tumors stratified according to tumor localization. (B) Scatter dot plot showing protein levels of ALDH1A1 in primary CRC tumors, treated and untreated, stratified according to tumor localization. (A and B) An unpaired T-test was applied to the log-transformed data to compare groups. The geometric mean is shown. CRC, colorectal cancer; Desc, descending; R, right. ns, p > 0.05; *, p ≤ 0.05.
(PDF)

S4 Fig ALDH1A1 levels are increased in colorectal cancer liver metastases. Scatter dot plot showing quantification of ALDH1A1 levels in colorectal cancer (CRC) tumors versus colorectal liver metastases (CRLM). Only patients from whom clinicopathological data was available were included. A paired T-test was applied to the log-transformed data to compare ALDH1A1 expression as continuous variable in CRC versus CRLM. *, p ≤ 0.05. The geometric mean is shown. CRC, colorectal cancer; CRLM, colorectal liver metastases.
(PDF)

S5 Fig ALDH1A1 expression is positively associated with expression of the nuclear receptor NR1I2 in liver metastases. The scatter plots show the association between the gene expression of ALDH1A1 and NR1I2 in primary colorectal cancer (CRC) tumor tissue (A-B) and colorectal cancer liver metastases (CRLM) (C-D). mRNA data was obtained from a large primary colon dataset [26] (A), a composite CRC cohort dataset [27, 28] (B), and two independent datasets containing expression data from CRLM [29](C) and [30] (D). (C-D) The color of each dot represents the expression of the KEGG pathway genes involved in xenobiotics metabolism. CRC, colorectal cancer; CRLM, colorectal liver metastases.
(PDF)

S6 Fig ALDH1A1 nuclear positivity identifies a group of poor-prognostic primary tumors and metastases. (A) ALDH1A1 nuclear staining was scored as either negative or positive. x10 microscope objective whole TMA tissue core (Scale bar: 100 μm) and x40 microscope objective inset (Scale bar: 50 μm) shown. (B) Kaplan-Meier curves showing differences in overall survival between ALDH1A1-positive and ALDH1A1-negative colorectal cancer tumors and liver metastases. ALDH1A1 status was based on the presence or absence of nuclear staining. Significance was tested using the log-rank test. CRC, colorectal cancer; CRLM, colorectal liver metastases; Neg, negative; Pos, positive.
(PDF)

S7 Fig ALDH1A1 positivity identifies a group of poor-prognostic primary tumors and metastases. (A) Kaplan-Meier curves showing the differences in overall survival between ALDH1A1-positive and ALDH1A1-negative primary colorectal cancer tumors stratified according to tumor location (right-sided versus left-sided). Significance was tested using the log-rank test. (B) Kaplan-Meier curves showing the differences in overall survival between ALDH1A1-positive and ALDH1A1-negative colorectal liver metastases stratified according to primary tumor location (right-sided versus left-sided). Significance was tested using the log-rank test. CRC, colorectal cancer; CRLM, colorectal liver metastases; L, left-sided; Neg, negative; Pos, positive; R, right-sided.
(PDF)

S1 Table Raw ALDH1A1 scorings data. (XLSX)
