# Supplementary figures and images for: ALDH1A1 expression is associated with poor differentiation, ‘right-sidedness’ and poor survival in human colorectal cancer
Source: PLoS One. 2018 Oct 11;13(10):e0205536. doi: 10.1371/journal.pone.0205536 (PMC6181398; doi:10.1371/journal.pone.0205536)

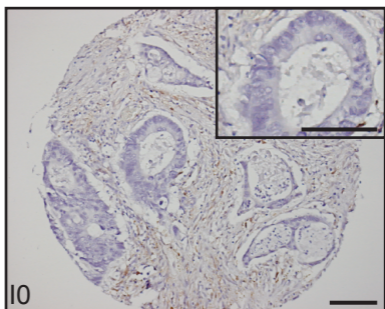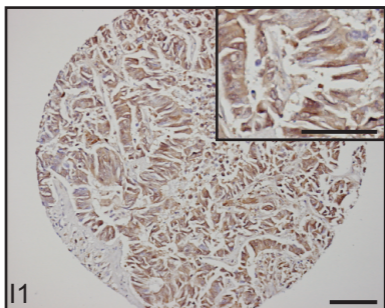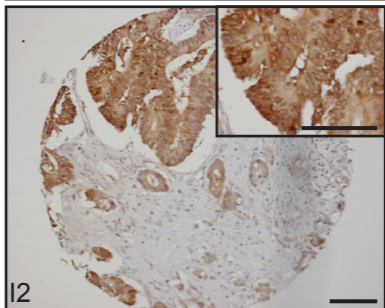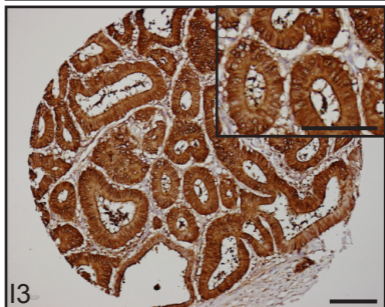

Supplement: S1 Fig — Images of the different staining intensities: 0 (negative), 1 (weak), 2 (moderate) or 3 (strong) are shown. I = intensity. Scale bar: 100 μm (x10 microscope objective). Inset shows higher magnification. (PDF) [file pone.0205536.s001.pdf]

A

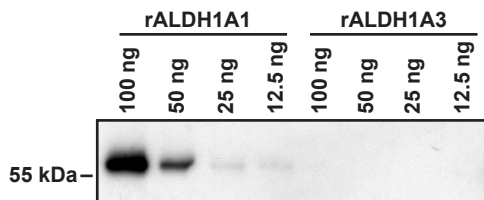

B

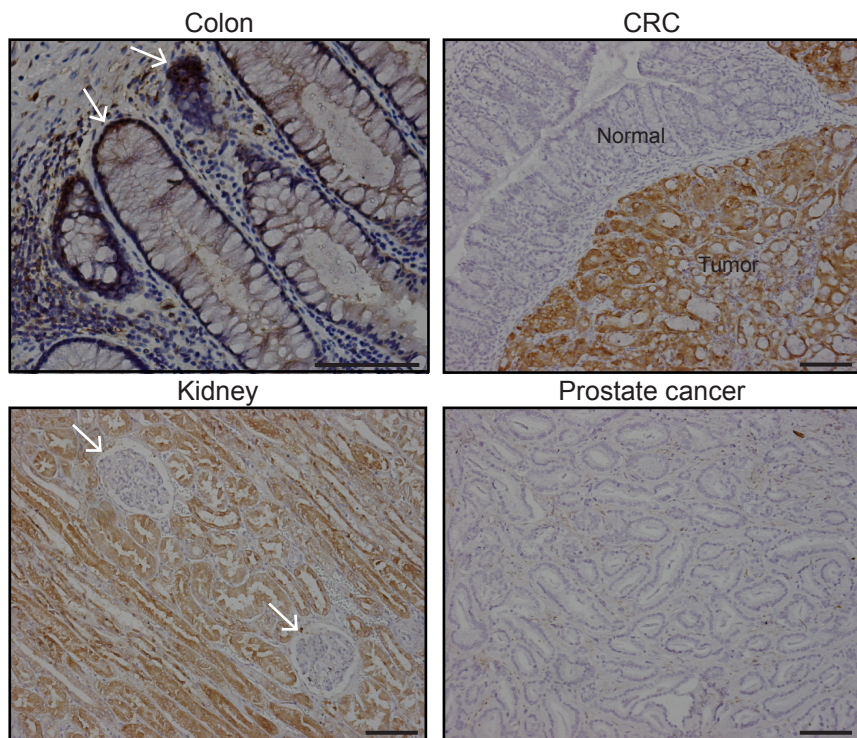

Supplement: S2 Fig — (A) Decreasing amounts of either recombinant ALDH1A1 (rALDH1A1) or rALDH1A3 were loaded to evaluate ALDH1A1 antibody specificity using Western blotting. (B) Immunohistochemical staining pattern of the antibody against ALDH1A1 in normal colon tissue, CRC tumor tissue, kidney and prostate cancer (negative control). White arrows indicate ALDH1A1 immunoreactivity in intestinal crypts (colon tissue) or absence of ALDH1A1 staining in kidney glomeruli. Scale bar: 100 μm (x10 microscope objective). (PDF) [file pone.0205536.s002.pdf]

A

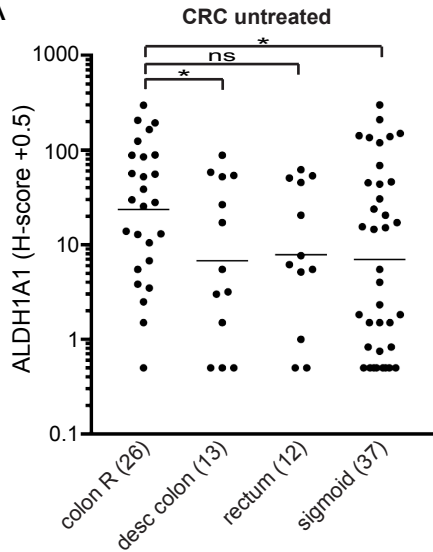

B

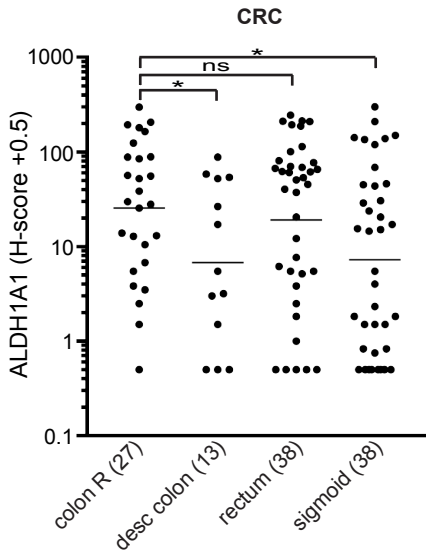

Supplement: S3 Fig — (A) Scatter dot plot showing protein levels of ALDH1A1 in untreated primary colorectal cancer (CRC) tumors stratified according to tumor localization. (B) Scatter dot plot showing protein levels of ALDH1A1 in primary CRC tumors, treated and untreated, stratified according to tumor localization. (A and B) An unpaired T-test was applied to the log-transformed data to compare groups. The geometric mean is shown. CRC, colorectal cancer; Desc, descending; R, right. ns, p > 0.05; *, p ≤ 0.05. (PDF) [file pone.0205536.s003.pdf]

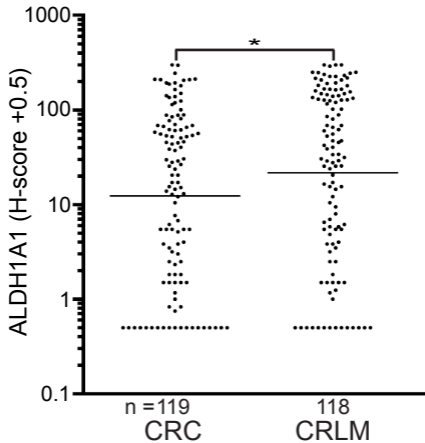

Supplement: S4 Fig — Scatter dot plot showing quantification of ALDH1A1 levels in colorectal cancer (CRC) tumors versus colorectal liver metastases (CRLM). Only patients from whom clinicopathological data was available were included. A paired T-test was applied to the log-transformed data to compare ALDH1A1 expression as continuous variable in CRC versus CRLM. *, p ≤ 0.05. The geometric mean is shown. CRC, colorectal cancer; CRLM, colorectal liver metastases. (PDF) [file pone.0205536.s004.pdf]

A

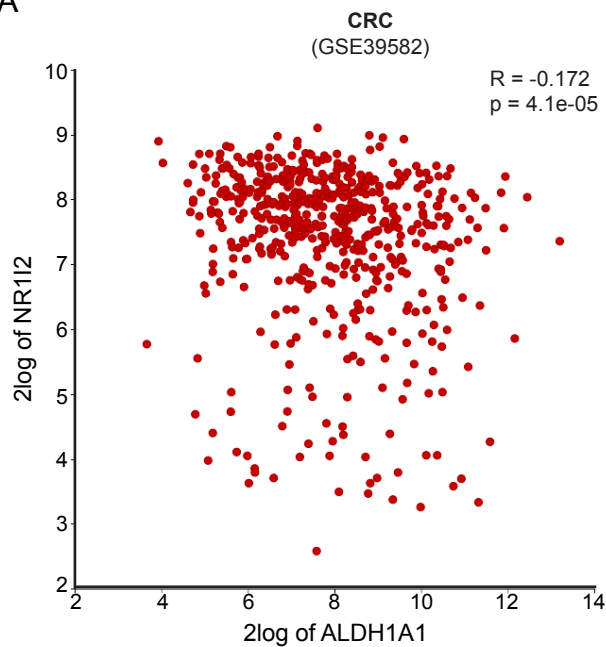

B

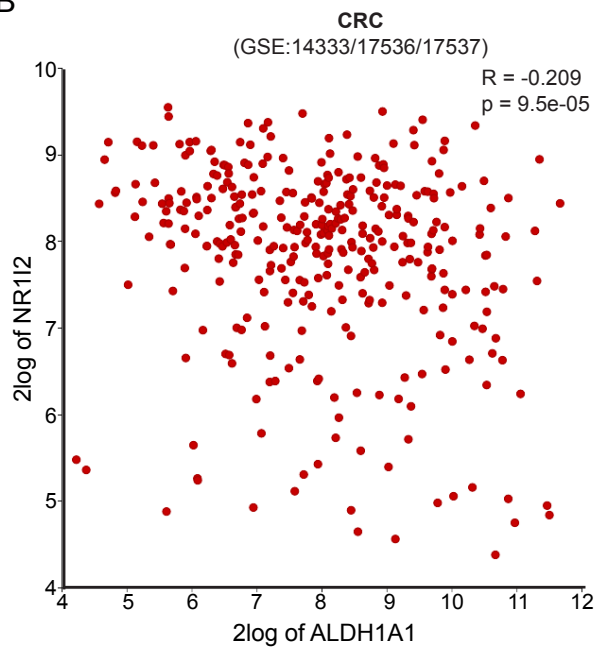

C

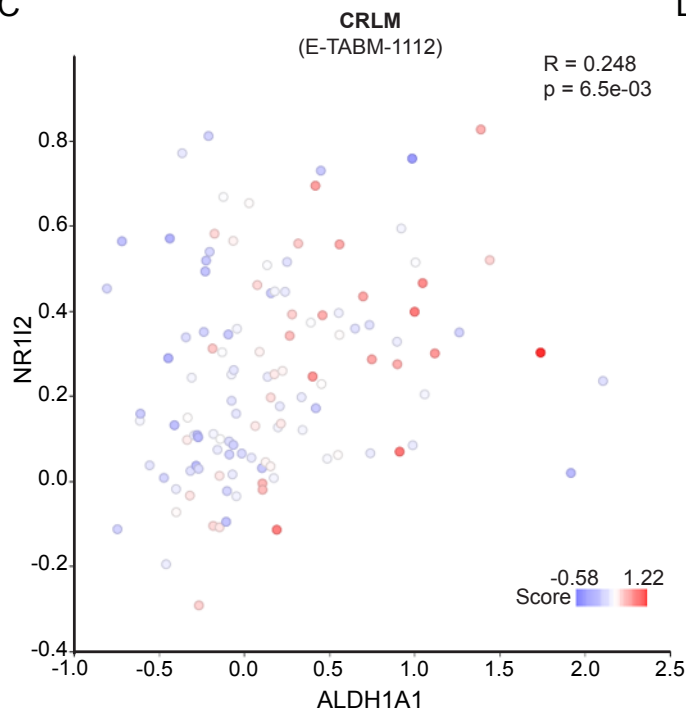

D

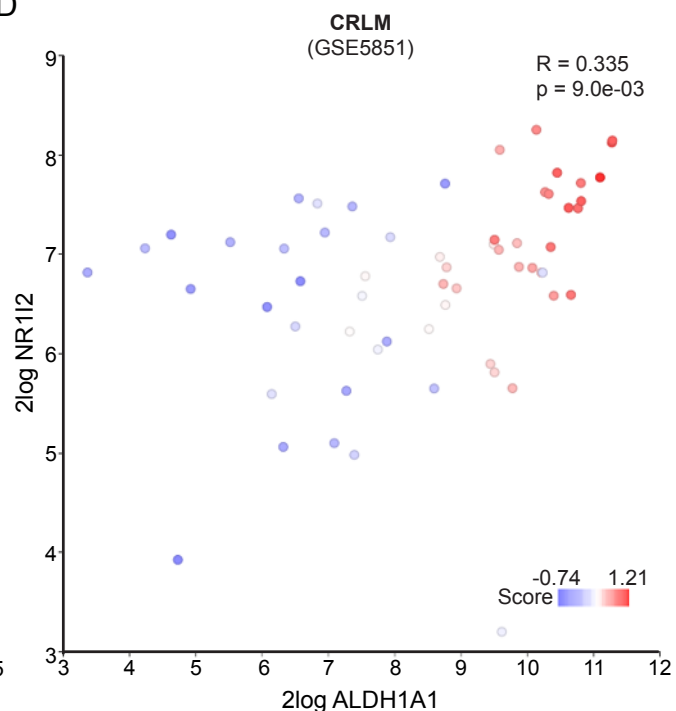

Supplement: S5 Fig — The scatter plots show the association between the gene expression of ALDH1A1 and NR1I2 in primary colorectal cancer (CRC) tumor tissue (A-B) and colorectal cancer liver metastases (CRLM) (C-D). mRNA data was obtained from a large primary colon dataset [26] (A), a composite CRC cohort dataset [27, 28] (B), and two independent datasets containing expression data from CRLM [29](C) and [30] (D). (C-D) The color of each dot represents the expression of the KEGG pathway genes involved in xenobiotics metabolism. CRC, colorectal cancer; CRLM, colorectal liver metastases. (PDF) [file pone.0205536.s005.pdf]

A

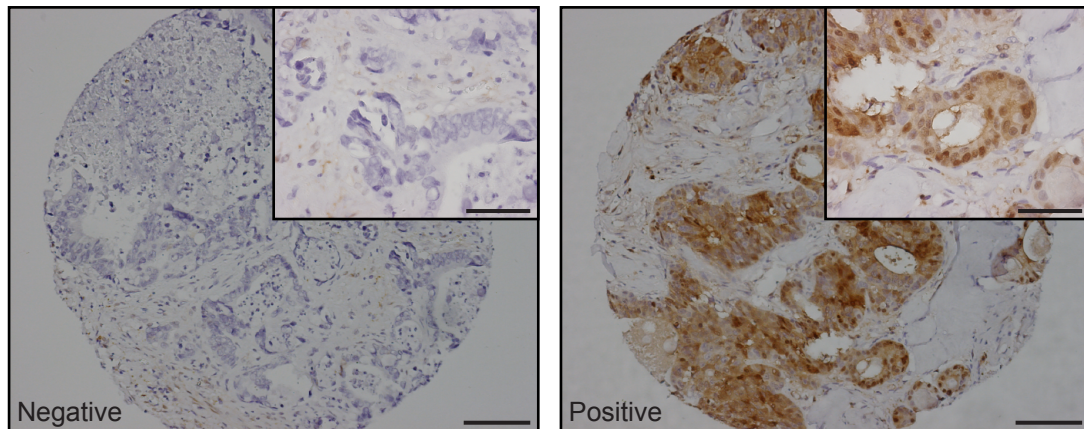

B

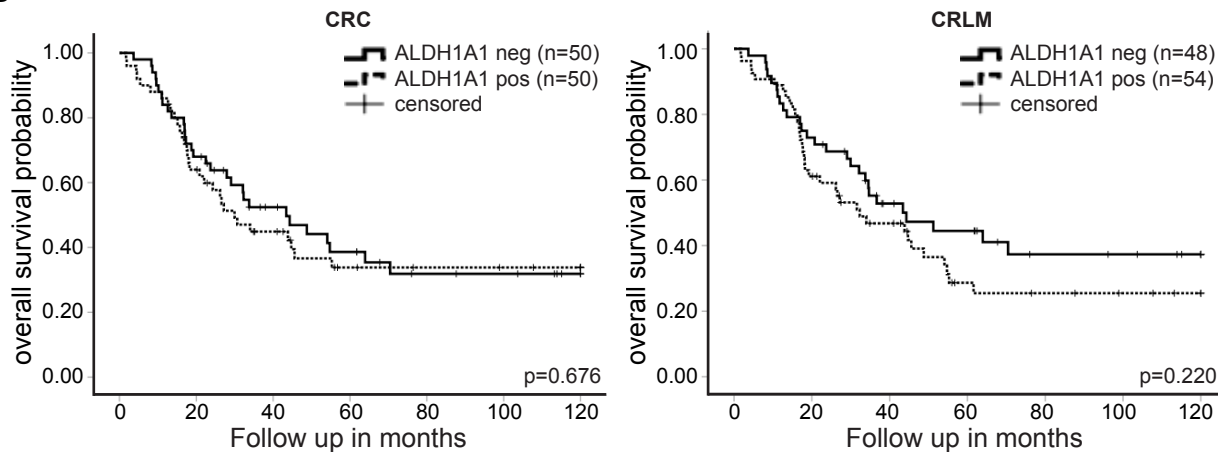

Supplement: S6 Fig — (A) ALDH1A1 nuclear staining was scored as either negative or positive. x10 microscope objective whole TMA tissue core (Scale bar: 100 μm) and x40 microscope objective inset (Scale bar: 50 μm) shown. (B) Kaplan-Meier curves showing differences in overall survival between ALDH1A1-positive and ALDH1A1-negative colorectal cancer tumors and liver metastases. ALDH1A1 status was based on the presence or absence of nuclear staining. Significance was tested using the log-rank test. CRC, colorectal cancer; CRLM, colorectal liver metastases; Neg, negative; Pos, positive. (PDF) [file pone.0205536.s006.pdf]

A

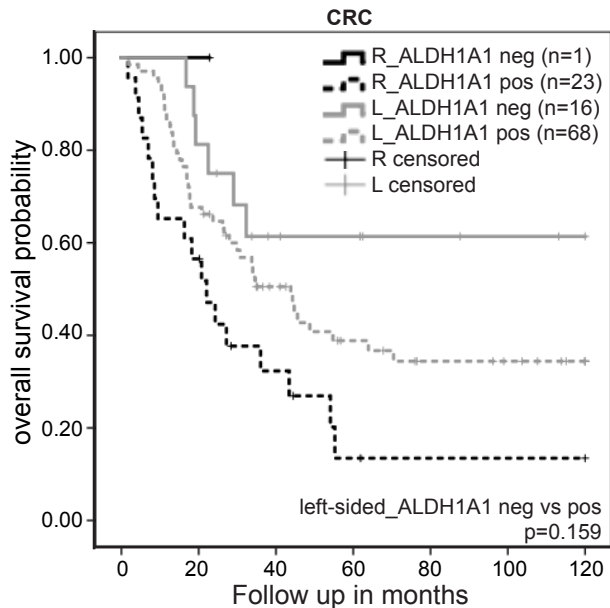

B

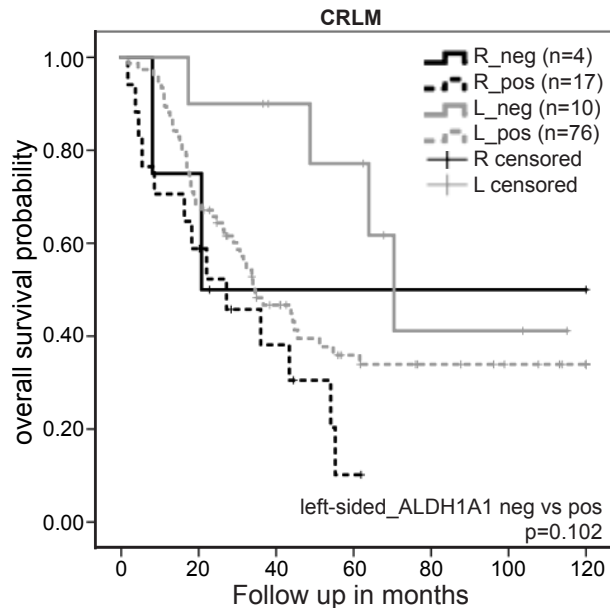

Supplement: S7 Fig — (A) Kaplan-Meier curves showing the differences in overall survival between ALDH1A1-positive and ALDH1A1-negative primary colorectal cancer tumors stratified according to tumor location (right-sided versus left-sided). Significance was tested using the log-rank test. (B) Kaplan-Meier curves showing the differences in overall survival between ALDH1A1-positive and ALDH1A1-negative colorectal liver metastases stratified according to primary tumor location (right-sided versus left-sided). Significance was tested using the log-rank test. CRC, colorectal cancer; CRLM, colorectal liver metastases; L, left-sided; Neg, negative; Pos, positive; R, right-sided. (PDF) [file pone.0205536.s007.pdf]
